# Use of an In Vitro Digestibility Approach to Assess *Bombyx mori* and *Camelina sativa* as Alternative Feed Ingredients for Poultry Species

**DOI:** 10.3390/vetsci12030277

**Published:** 2025-03-15

**Authors:** Yazavinder Singh, Antonella Dalle Zotte, Marco Cullere, Pipatpong Chundang, Penpicha Kongsup, Attawit Kovitvadhi

**Affiliations:** 1 Department of Animal Medicine, Production and Health, University of Padova, Agripolis, Viale dell’Università 16, Legnaro, 35020 Padova, Italy; yazavinder.singh@unipd.it (Y.S.); marco.cullere@unipd.it (M.C.); penpicha.kong2@gmail.com (P.K.); 2 Department of Physiology, Faculty of Veterinary Medicine, Kasetsart University, Bangkok 10900, Thailand; pichandang@gmail.com (P.C.); attawitthai@gmail.com (A.K.); 3 KU VET INNOVA NUTRICARE Co., Ltd., Faculty of Veterinary Medicine, Kasetsart University, Bangkok 10900, Thailand

**Keywords:** chicken, quail, gastrointestinal tract, enzyme, silkworm, camelina

## Abstract

The present study investigated the in vitro digestibility of diets for broiler chickens (Ross308) and broiler and laying Japanese quails (*Coturnix japonica*). The diets contained unconventional feedstuffs such as silkworm (*Bombyx mori*) meal (SWM) and meals obtained from different *Camelina sativa* lines (Pearl and Alan, characterized by reduced linoleic acid and glucosinolates content, respectively). An in vitro technique was tested in order to assess its potential for replacing in vivo studies. To test this, the digestive tracts of fifty broiler chickens and four hundred Japanese quails were sampled to extract digestive enzymes to be used for in vitro digestibility assessments, including dry matter digestibility (DMd), organic matter digestibility (OMd), and crude protein digestibility (CPd). Diets including SWM exhibited comparable digestibility values to those of Control diet for broiler chickens, highlighting its potential as a valuable protein source in poultry nutrition. The study also found strong correlations between DMd and OMd (*p* < 0.01), indicating a logical relationship in nutrient breakdown. A 5% or 10% inclusion of the two camelina lines in the diet for broiler quails did not significantly alter the digestibility parameters (*p* ≥ 0.05), whereas in laying quails, diets with a 15% inclusion level of camelina resulted in a significant difference in digestibility (*p* < 0.05). Specifically, oil diets provided the best outcomes, while the diet Pearl Spring 15 showed the lowest DMd, CPd, and OMd (*p* < 0.05). Overall, results from the present study indicate that the tested alternative feedstuffs (SWM and camelina) have a good potential for poultry feed formulations. In addition, the tested in vitro technique was shown to be more suitable to predict the digestibility of single feedstuffs (i.e., SWM) rather than complete diets, which is consistent with the existing literature. For this reason, this in vitro technique is not adequate to replace in vivo digestibility experiments.

## Simple Summary

The present study assessed the in vitro digestibility of alternative feed ingredients (silkworm meal, camelina meal) in broiler chicken and Japanese quail diets using an in vitro method exploiting digestive enzymes collected at a commercial slaughterhouse. The results showed similar trends and findings to those from in vivo trials involving the same animal species and testing the same diets/ingredients. Moreover, the method was revealed to be effective in screening single feedstuffs before being tested in animal studies. In contrast, the study also highlighted the limitations of this in vitro technique in predicting the digestibility of complete diets, mainly with regards to the absolute values of digestibility coefficients, ultimately resulting in underestimation. In conclusion, the tested in vitro assay cannot be substituted for animal-based tests for accurate diet evaluation.

## 1. Introduction

Soybean meal and fish meal are primary protein sources in poultry diets, with fish meal particularly valued for its well-balanced amino acid profile, high digestibility, and palatability. However, many feed manufacturer avoid using fish meal in poultry feed due to its higher variability in quality, potential risk of cross-contamination, and environmental concerns associated with the sustainability of the practice [1]. Consequently, fish meal is generally replaced by soybean meal, despite its less balanced amino acid profile. However, soybean cultivation has other disadvantages, since it requires significant land and water resources, as well as pesticides, and the production for feed is also in competition with that for food. For the above-mentioned reasons, research in the last decade has been focusing on exploring alternative protein sources for animal feeding that possess remarkable sustainability attributes.

In this regard, insects are promising candidates to satisfy the nutritional needs of poultry species. Among them, the silkworm (*Bombyx mori*) is a Lepidopteran species primarily reared for silk production in the textile industry. The pupae constitute a major by-product of sericulture, and, while utilized as a food source in certain regions, they are often discarded, leading to the loss of valuable nutrients and contributing to environmental concerns [2]. From a nutritional perspective, silkworm meal (SWM) is rich in protein, with a protein content ranging from 52% to 80% (dry matter basis). It also provides a substantial lipid fraction, varying between 10% and 30% (dry matter basis), which includes healthy lipids, mainly polyunsaturated fatty acids of the omega-3 series. However, SWM also contains a bioactive compound, 1-deoxynojirimycin (1-DNJ), which could represent an issue for animal nutrition. In fact, 1-DNJ acts as a potent intestinal α-glucosidase inhibitor. Its mechanism of action involves competitive inhibition of specific glycosidase enzymes by mimicking the structural properties of normal carbohydrate substrates such as D-glucose and D-mannose [3]. As a result, 1-DNJ can interfere with glycogenolysis, glycoprotein processing, and saccharide hydrolysis, necessitating a thorough evaluation of its implications when incorporating SWM into poultry diets. Furthermore, SWM contains chitin, a structural polysaccharide found in the exoskeletons of arthropods and other organisms. Research indicates that chitin can negatively impact nutrient absorption in poultry by forming complexes with essential nutrients, particularly proteins and lipids, thereby reducing their digestibility [4]. Given these factors, while SWM presents potential as an alternative protein source in poultry nutrition, its dietary inclusion requires careful assessment to optimize nutrient utilization and mitigate any adverse effects.

Additionally, there are oil seed crops from which oil is extracted and cake is discarded or used as fertilizer, as it contains antinutritional compounds. Among them, *Camelina sativa* stands out as a promising feedstuff due to its sustainability. This crop has a relatively short growth cycle, requires minimal agricultural inputs, and tolerates common *Brassica* pests and diseases, thereby necessitating fewer treatments. Camelina meal contains around 35% protein content and fat ranging from 5% to 28% of residual oil, particularly rich in essential omega-3 fatty acids. Moreover, the use of improved lines with reduced antinutritional compounds (such as such as glucosinolates, phytic acid, sinapine, trypsin inhibitors, condensed tannins, and erucic acid) could facilitate their successful incorporation as raw feed material for livestock species, particularly poultry [5,6,7,8].

Determining in vivo digestibility is an effective method for assessing the nutritional value of poultry diets and evaluating digestive tract function, providing valuable information for feed evaluation and gastrointestinal health status. However, this technique is well-known for its disadvantages, including the use of animals, cost considerations, and the fact that it is time-consuming.

It is also accepted that some in vitro digestibility protocols correlate well with in vivo counterparts, with the advantages being that in vitro studies are easier to conduct, faster, and cheaper and do not directly involve animals. This technique has been successfully used to screen or assess the digestibility of possible feed ingredients in different animal species and humans. Generally, in vitro digestibility is investigated using commercially available enzymes or enzymes extracted from swine to represent poultry species [9,10,11,12,13,14,15]. However, utilizing crude enzymes extracted from the same animal species would be preferable for such feed screening tests. Therefore, the present study aimed to explore the potential of an in vitro digestibility technique using crude enzymes extracted from broiler chickens and quails to preliminarily screen novel feedstuff candidates for poultry. This involved a critical evaluation of the tested in vitro method, both in absolute terms and by comparing results with in vivo trials, when available.

## 2. Materials and Methods

### 2.1. Feed Samples

Feed samples containing camelina cakes were used in the in vitro digestibility trial for quails as part of the project “Agronomic and genetic improvement of camelina (*Camelina sativa* (L.) Crantz) for sustainable poultry feeding and healthy food products (PRIN “Argento” project: Prot. 2017LZ3CHF, https://site.unibo.it/progetto-argento/en (accessed on 17 September 2024)). Within the project, different experiments on broiler and laying quails (*Coturnix japonica*) were designed in order to assess the dietary incorporation of camelina (*Camelina sativa*) cake and oil as sustainable feed ingredients for poultry. Different camelina lines, sowing dates, and inclusion levels were assessed in the project. For the present in vitro digestibility research, the diets from two experiments performed as part of the Argento project were selected: the first one studied the inclusion of different lines (Alan vs. Pearl), sowing dates (spring vs. autumn), and inclusion levels (0% vs. 5% vs. 10%) of camelina cake in the diets for growing quails. The second one assessed the inclusion of different sowing dates (spring vs. autumn) of different camelina products (15% cake, or oil as 100% conventional oil replacement) in the diets for laying quails.

In addition, the current in vitro digestibility study considered a single feedstuff and the experimental diets of another in vivo study on broiler chickens: the aim was testing the dietary incorporation of 4% silkworm (*Bombyx mori*) chrysalis meal (SWM) at different phases (starter phase: 1–10 days, or grower-finisher phase: 11–42 days) of the chicken growth cycle [6]. Specific information about the experimental diets and the SWM involved in the present experiment are presented in Table 1.

### 2.2. Chemicals

The following chemicals were purchased from Sigma Chemical Co.: 3,5-dinitrosalicylic acid (C_7_H_4_N_2_O_7_, 97%), acetone (≥99%), carboxymethylcellulose sodium salt (average Mw ≅ 90,000), casein (technical grade), chitin (shrimp shell, purified powder for chitinase), chloramphenicol (99.5%), *D*-(+)-glucose monohydrate (C_6_H_12_O_6_.H_2_O), *D*-(+)-maltose monohydrate (C_12_H_22_O_11_·H_2_O), ethanol (99%), Folin–Ciocalteu reagent (2N), hydrochloric acid (HCl, 37%), *L*-tyrosine (≥99%), potassium sodium tartrate tetrahydrate (99%), sodium carbonate (Na_2_CO_3_), sodium chloride (NaCl, ≥99%), sodium hydroxide (NaOH, 99%), sodium phosphate dibasic (Na_2_HPO_4_, ≥99%), sodium phosphate monobasic dihydrate (NaH_2_PO_4_.·H_2_O, ≥99%), starch (soluble, ACS for analysis), and trichloroacetic acid (C_2_HCl_3_O_2_, ≥99%).

### 2.3. Digestive Enzyme Extraction and Enzyme Activity

**Figure 1:**
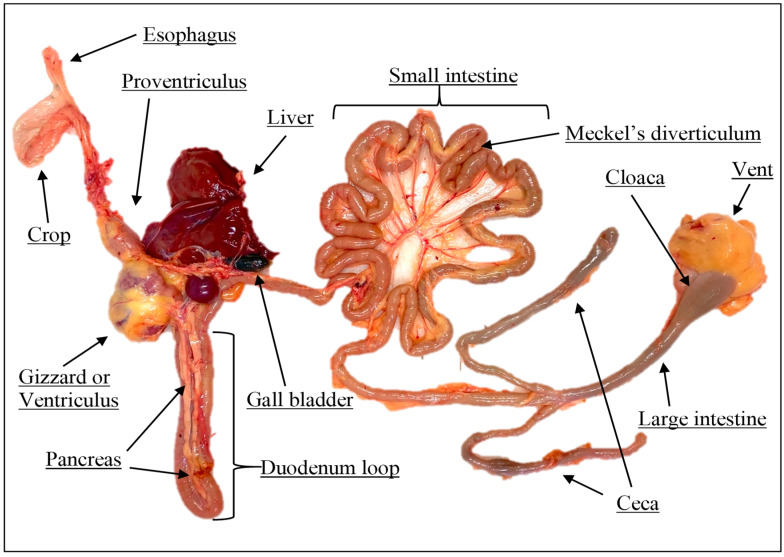
Schematic view of gastrointestinal tract of poultry species.

The digestive enzyme extraction method was adapted starting from the method published by Kovitvadhi et al. [13]. At a commercial slaughterhouse, during the evisceration process, digestive tracts were obtained from fifty 42-day-old broiler chickens (Ross308) and four hundred 35-day-old Japanese quails (*Coturnix japonica*) (Figure 1) and placed in sterile plastic bags. Afterwards, they were immediately placed on ice to preserve the enzyme activity. The collected tracts were then transferred to the laboratory LabCNX of the Department of Animal Medicine, Production and Health (MAPS) of the University of Padova, Italy. In the laboratory, the digestive organs, such as the proventriculus, pancreas, and duodenum, were extracted and washed with 0.2 M phosphate buffer solution (PBS; pH 7). The gastric mucosa, pancreas (whole organ), and duodenal mucosa were collected. Samples of each organ were pooled and homogenized in 0.2 M PBS (pH 7, 1:5 *w*/*v*) on ice, followed by centrifugation at 4000 rpm at 4 °C for 50 min. The supernatant, containing crude enzyme extract (CEX), was stored at −40 °C for further analyses.

Pepsin activity (pH 3) was determined in the gastric mucosa CEX, whereas amylase and cellulase activity (pH 7) were evaluated for the pancreas and duodenal mucosa according to the procedure described by Kovitvadhi et al. [13], with modifications to confirm the enzyme activity of the CEX before beginning the experiments. Enzyme activity in the CEX obtained from different organs was analyzed in sextuplicate. For pepsin, the substrate (1% casein dissolved in 50 mM PBS, pH 8) and gastric mucosa CEX were mixed in 0.2 M PBS at pH 3 and incubated at 40 °C for 30 min. After incubation, trichloroacetic acid (TCA, 10% *v*/*v*) was added to stop the enzyme reaction, followed by centrifugation at 4000 rpm for 20 min at 4 °C. The supernatant was mixed with 0.5 M Na_2_CO_3_ and 5 M Folin–Ciocalteu reagent, and the absorbance of the free amino acids was recorded at a wavelength of 660 nm, using the L-tyrosine standard curve as a reference. Soluble starch (5% *w*/*v*) was used as a substrate for determining the amylase activity of pancreatic and duodenal mucosa CEX (1:1 *v*/*v*) at pH 7. After 15 min of incubation at 37.5 °C, 3,5-dinotrosalicylic acid (1% *v*/*v*) was added, followed by boiling at 100 °C for 5 min. The mixture was allowed to cool down at room temperature, distilled water (1.2 mL) was added, and the absorbance was measured at 540 nm, using the maltose standard curve as a reference. Carboxy methyl cellulose (CMC, 1% *w*/*v*) was used as substrate for determining the cellulase activity of pancreatic and duodenal mucosa CEX (1:1 *v*/*v*) at pH 7. The mixture was incubated at 40 °C for 30, and 3,5-dinotrosalicylic acid (1% *v*/*v*) was added, followed by boiling at 100 °C for 5 min. The mixture was allowed to cool at room temperature, after which distilled water (1.2 mL) was added, and the absorbance was measured at 540 nm, using the glucose standard curve as a reference.

### 2.4. In Vitro Digestibility Assay

A two-step in vitro digestibility was carried out for each substrate, including a blank reaction, using an approach based on the methods outlined by Kovitvadhi et al. [13,15]. The protocol for the in vitro digestibility experiment was the same for the two poultry species. One gram of substrate (experimental diets from experiment 1, 2, and 3, or SWM; presented in Table 1) was mixed with 15 mL of 0.1 M PBS buffer (pH 6) and 2 mL of 0.2 N HCl. The pH was adjusted to 3 by adding 1 N HCl or 1 N NaOH. Afterwards, 2 mL of gastric mucosal CEX was added to 0.2 mL of 5% chloramphenicol. The mixture was incubated at 37.5 °C for 2 h with continuous shaking at 200 rpm. After incubation, 2 mL of 0.2 M PBS (pH 7) and 1 mL of 0.6 N NaOH were added, and then the pH was adjusted to 7 with 1 N HCl or 1 N NaOH. The mixture of pancreatic and duodenal mucosal CEX (1:1 *v*/*v*, 1 mL) was added before incubation at 40 °C for 4 h with continuous shaking at 200 rpm. After incubation, 10 mL of 10% TCA was added and placed at room temperature for 30 min to stop the enzyme reaction. The mixtures were stored at −20 °C, and following day they were thawed, and sediments were filtered (pore diameter: 100 um), then rinsed three times with distilled water, ethanol, and acetone, respectively. The obtained in vitro digestibility residue was dried at room temperature, packed, and sent to the Department of Physiology, Faculty of Veterinary Medicine, Kasetsart University, Thailand, for chemical analyses.

### 2.5. Chemical Analysis

The silkworm (*Bombyx mori*) meal and the experimental diets for broiler chickens, broiler quails, and layer quails were analyzed in duplicate for dry matter (DM; method no. 930.15), crude protein (CP; method no. 984.13), ether extract (EE; method no. 2003.05), crude fiber (CF; method no. 962.09), and ash (method no. 942.05) content, following Association of Official Analytical Chemists (AOAC, 2006) methods. A nitrogen-to-protein conversion value of 4.76 was used for SWM to represent crude protein content [16]. Organic matter (OM) was obtained by calculating the difference: DM—ash. For in vitro digestibility residues, DM, CP, ash, and OM contents were determined by following the above-mentioned methods.

### 2.6. Statistical Analysis

Statistical analysis was performed in R, using the Rcmdr Package [17]. The normal distribution and homogeneity of variance of the data were confirmed by Shapiro–Wilk test and Levene’s test, respectively. One-way ANOVA was performed to evaluate the differences in DMd, OMd, and CPd among substrates as fixed factors, and Duncan’s new multiple range test was used as a *post hoc* test. Pearson’s correlation coefficient analysis was performed to determine the chemical composition and percentages of DMd, OMd, and CPd in the substrates. Statistical significance was defined as *p* < 0.05.

## 3. Results

The chemical composition of different diets for broiler chickens containing SWM, as well as the results concerning the in vitro digestibility, are presented in Table 2, Table 3, and Table 4. The experimental diets included Starter, Grower, and Finisher versions. The crude protein content was balanced among the diets, which had been designed to be isonitrogenic and isoenergetic. The ether extract content was slightly higher in the SWM diets compared to the Control diets for each growth phase. The in vitro digestibility results highlighted that the Control Finisher diet displayed lower digestibility than the SWM Starter diet (*p* < 0.001), while the other groups had intermediate results. The same outcome was observed for the OM digestibility (*p* < 0.001). CP was better digested in the SWM Starter and Finisher diets compared to the Control Grower and Control Finisher diets (*p* < 0.001), while the Control Starter and SWM Grower diets exhibited intermediate values. Correlation coefficient analysis showed that DMd had a strong and positive correlation with the DM diet (r = 0.894), CP diet (r = 0.914), and EE diet (r = 0.909), and a lower, yet significant, positive correlation with the Ash diet (r = 0.411). In contrast, a negative correlation was observed with the CF diet (r = −0.416). The correlations of OMd with the DM diet, CP diet, EE diet, Ash diet, and CF diet were almost overlapping with the above-mentioned correlations for DMd. CPd was significantly correlated with all the considered digestibility traits, but the relationships were not as robust as those described for DMd and CPd.

Table 5 presents the chemical composition of the diets for broiler quails containing different lines, sowing dates, and inclusion levels of *Camelina sativa* cake, while Table 6 and Table 7 show the resulting in vitro digestibility outcomes and related correlation coefficients, respectively. Unlike the previous in vitro digestibility test, the Control diet displayed comparable outcomes with all treatment groups, as no significant differences were observed. The digestibility coefficients provided different information compared to the previous trial: specifically, the digestibility coefficients showed non-significant correlations with dietary components (DM diet, Ash diet, CP diet, EE diet, CF diet).

The results concerning the inclusion of camelina cake (same inclusion level, but different lines and sowing dates) or oil (Pearl line, different sowing dates) in the diets for laying quails are depicted in Table 8 (feed), Table 9 (in vitro digestibility), and Table 10 (correlation coefficients). The experimental diets were well balanced in terms of the CP content, which was a formulation target. The digestibility results highlighted that feed containing camelina oil (both Spring and Autumn sowing dates) and cake (Alan Spring) had the highest DMd (*p* < 0.05), CPd (*p* < 0.01), and OMd (*p* < 0.05), while the Pearl Spring diet displayed the worst results. Looking at the correlation coefficients, DMd and OMd were significantly correlated with the EE diet (r = −0.355 and r = −0.367, respectively) and OMd with the CF diet (r = 0.310) and Ash diet (r = 0.310), even though the coefficients were low in absolute terms. In addition, CPd was negatively correlated with the CP diet (r = −0.539), EE diet (r = −0.486), and CF diet (r = −0.154), also in this case with moderate to low coefficients.

## 4. Discussion

It is widely accepted that in vivo animal studies are ideal to provide accurate information on nutrient utilization, which can reliably predict performance. In contrast, in vitro methods (especially for poultry species) cannot fully replace in vivo studies. However, the development of in vitro protocols has garnered significant research attention for several reasons, including the short time required for evaluations, minimal personnel needs, low economic investment, and the ability to conduct assays in virtually any laboratory [18]. Additionally, the use of in vitro studies addresses ethical concerns associated with animal use in scientific research and avoids various factors (such as genetics, environmental conditions, potential disease occurrence, and management practices) that can affect in vivo digestibility results. The development of specific in vitro digestibility methods for poultry has been relatively recent, as historically, in vitro data for monogastric species, such as pigs, were adapted for poultry studies [11]. Given the significant differences in digestive tract anatomy between poultry and pigs (such as the presence of a crop and gizzard in poultry and a well-developed hindgut in pigs), implementing in vitro digestibility protocols specifically designed for poultry is advisable. This approach is particularly pertinent considering the importance of poultry in global animal production. [19]. An additional beneficial aspect of in vitro enzymatic digestion protocols, as adopted in the present study, is the use of crude digestive enzymes extracted from the same animal species and/or individuals intended for future in vivo studies. This method enhances the potential for better correlation coefficients between in vitro and in vivo results. [20]. However, this approach presents a possible drawback: sampling digestive organs from slaughtered animals means that enzyme concentration and activity can be affected by the chemical composition of the diets the animals were consuming. This variability can influence observed enzymatic activity and, consequently, affect the outcomes of in vitro digestibility assays utilizing the extracted enzymes. In fact, the results presented in Table 11 highlight that the crude enzyme extracts from the proventriculus, pancreas, and duodenum of 42-day-old broiler chickens vs. 35-day-old quails showed remarkable differences in amylase activity (11.8 vs. 1.01 U/min/mL for broiler chickens and broiler quails, respectively).

The first consideration arising from the results obtained with the present in vitro digestibility assay is that, in absolute terms, the digestibility of DM, OM, and CP showed lower values compared to the usual in vivo studies for the same species for complete diets. For example, recent research testing the dietary inclusion of 12.5% SWM meal (full-fat) in the diet for broiler quails showed approximately 51% DM, 70% CP, and 54% OM digestibility [21], which are remarkably higher than the values overall observed in the present research. This was only partly surprising, as experimental diets are complex food matrices composed of various ingredients, each with distinct nutritional and antinutritional properties. This complexity is particularly relevant because in vitro digestibility assays are static systems, unlike the dynamic gastrointestinal tract (GIT) of an animal, which involves feed–physiology interactions and responses to ingested feed. In vitro systems lack this dynamic interaction, potentially limiting their ability to accurately simulate the digestive process. In fact, enzymatic in vitro assays can be hindered by non-digestible constituents, such as plant cell walls or insoluble dietary fibers, which may increase in the diet compared to the raw materials. The swelling and viscosity behavior of these components impact macronutrient digestion and absorption by reducing enzyme diffusion, thus limiting their action [22]. In addition, enzyme dilution and substrate (diet) concentration play a pivotal role in determining the level of hydrolysis and absorption of nutrients. Increasing substrate concentration can enhance the rate of hydrolysis up to a certain point. Once all available enzymes are saturated, any further increase in substrate concentration will not affect the hydrolysis rate, as the enzymes are already operating at their maximum capacity. This suggests that increasing the enzyme concentration or decreasing the dilution rate, as well as reducing the quantity of dietary substrate used in the test, may be considered in future research.

Literature data indicate that, for the all the above-mentioned reasons, dietary ingredients should be considered individually when performing in vitro digestibility experiments, and that, at present, this technique could be successfully used to screen the potential of new emerging feedstuffs, but without claiming to be a reference for feed formulations [18]. An example in this sense is a recent publication [15] that successfully screened 18 feed ingredients intended for different poultry species with very diverse nutritional profiles (DM digestibility was between 40% and 72%, and CP digestibility ranged from 15% to 47%). Nevertheless, there are also cases in which also certain feedstuffs, such as lupin meal, have been demonstrated to be unsuitable for in vitro digestion models [23]; this is attributable to possible interferences in the digestion process due to the presence of antinutritional factors in the tested raw material.

The adequacy of in vitro digestibility studies for single feed ingredients rather than complete diets emerges in the present research too: in fact, when the results of the in vitro digestibility of SWM alone are considered (Table 2), a different scenario appears than that for experimental diets: the SWM digestibility values for DM (48.9%), OM (46.5%), and CP (47.7%) were consistent with data published by Kovitvadhi et al. [15], in which the in vitro digestibility values of SWM (broiler chicken) for DM, OM, and CP were 67%, 65% and 41%, respectively. Overall, the digestibility results were also consistent with other studies where single ingredients were tested for broiler chickens and meat-type ducks [13,14], as well as black-meat chickens and quail [15].

Despite the evident limitations of the in vitro digestibility protocol tested in the present trial regarding the quantification, in absolute terms, of DMd, CPd, and OMd, the observed dietary effects were consistent with existing in vivo results. Specifically, the same diets including the SWM for broiler chickens were also tested in a trial of broiler chickens, where performance traits were considered [6]. The results indicated that birds displayed similar live weight, average daily gain, feed intake, and FCR, thus suggesting the reliability of the results displayed in Table 2, whereas SWM diets showed comparable digestibility values with Control diets.

In contrast, experimental diets containing *Camelina sativa* cake and subjected to in vitro digestion exploiting crude enzyme extract from 35-day-old quails (Table 6 and Table 9) provided different indications: on the one hand, the incorporation of different camelina lines (Alan vs. Pearl), sowing dates (spring vs. autumn), and inclusion levels (0% vs. 5% vs. 10%) did not affect the DMd, CPd, and OMd. On the other hand, the incorporation of 15% camelina cake of the Pearl cultivar digested in vitro using the same crude enzyme extract (35-day-old quails) displayed a worse DMd, CPd, and OMd than the Control diet. Camelina is an alternative emerging oilseed crop for poultry dets, thanks to different interesting features including its tolerance to cold climates and drought, the low requirement for pesticides and fertilizers, as well as a remarkable *n*-3 FA content, as well as antioxidants [7]. However, camelina also contains glucosinolates, sinapine, condensed tannins, trypsin inhibitors, and phytic acid, which are antinutritional factors that, at certain ingestion levels, can have negative effects on digestibility by poultry, and thus performance [24]. Glucosinolates, in particular, once hydrolyzed in the animal intestine, generate some toxic products (isothiocyanates, thiocyanates, and nitriles) which are known to interfere with thyroid and liver function, and which can have negative effects on animal health and performance [25].

The results of the present study indicate that the Pearl Spring 15 diet had worse in vitro digestibility outcomes, while the same was not observed for other camelina cultivars with different sowing dates, even considering the same inclusion level. This was not surprising, and is attributable to different aspects: while the Alan cultivar was genetically engineered to have a low glucosinolate content [7], the Pearl cultivar was not. In addition, differences in the sowing season are known to produce seeds with different chemical characteristics [26], and thus also different glucosinolate contents [27]. Last but not least, the inclusion level is a key factor in determining digestibility, as it determines also the absolute amounts of antinutritional factors present in the diet and thus ingested by the animal, in this case quail. The above-mentioned hypotheses found confirmation in a trial studying the incorporation of different *Camelina sativa* cakes (15% inclusion rate) into diets for broiler quails [7], where the Pearl group exhibited worse live performance than the Control group.

## 5. Conclusions

The results of the current in vitro digestibility assay on SWM confirm its effectiveness as a preliminary tool for screening alternative feedstuffs prior to conducting in vivo studies on animals. However, the in vitro protocol used for poultry species also highlights its limitations in predicting the digestibility of complete diets. Specifically, the absolute values of digestibility coefficients (DMd, CPd, OMd) were lower than those reported in the literature from in vivo studies of the same species. Despite this, the experimental outcomes aligned with those observed in in vivo trials where the same or similar dietary treatments were tested on the same poultry species. For example, diets containing SWM were assessed in a trial evaluating broiler chickens’ performance traits. The results showed that the birds exhibited similar live weight, average daily gain, feed intake, and feed conversion ratio, suggesting the reliability of the in vitro results (SWM did not negatively affect DMd, CPd, or OMd). Overall, the present in vitro test is recommended for evaluating individual novel feedstuffs, while in vivo digestibility studies should be reserved for complete diets.

## Figures and Tables

**Table 1:** Information regarding diets and silkworm meal (SWM).

Acronyms	
Samples from experiment 1—Broiler chicken diets containing SWM	
Control Starter	Control diet (1–10 days of age)
Control Grower	Control diet (11–25 days of age)
Control Finisher	Control diet (26–42 days of age)
SWM Starter	Diet containing 4% defatted SWM (1–10 days of age)
SWM Grower	Diet containing 4% defatted SWM (11–25 days of age)
SWM Finisher	Diet containing 4% defatted SWM (26–42 days of age)
SWM (Pure)	Defatted SWM (100%) (feedstuff)
Samples from experiment 2—Broiler quail diets containing camelina (*Camelina sativa*) cake	
Control	Control diet
Alan Autumn 5	Diet containing 5% Alan line sowed in autumn
Alan Autumn 10	Diet containing 10% Alan line sowed in autumn
Alan Spring 5	Diet containing 5% Alan line sowed in spring
Alan Spring 10	Diet containing 10% Alan line sowed in spring
Pearl Autumn 5	Diet containing 5% Pearl line sowed in autumn
Pearl Autumn 10	Diet containing 10% Pearl line sowed in autumn
Pearl Spring 5	Diet containing 5% Pearl line sowed in spring
Pearl Spring 10	Diet containing 10% Pearl line sowed in spring
Samples from experiment 3—Layer quail diets containing camelina (*Camelina sativa*) cake or camelina oil	
Control	Control diet
Alan Autumn 15	Diet containing 15% Alan line sowed in autumn
Alan Spring 15	Diet containing 15% Alan line sowed in spring
Pearl Autumn 15	Diet containing 15% Pearl line sowed in autumn
Pearl Spring 15	Diet containing 15% Pearl line sowed in spring
Pearl Spring oil	Diet containing 0.39% Pearl line sowed in spring
Pearl Autumn oil	Diet containing 0.39% Pearl line sowed in autumn

**Table 2:** Proximate composition (%) of samples from experiment 1—broiler chicken diets containing silkworm meal (SWM).

	As Is					Dry Matter			
	Moisture	Ash	CP	EE	CF	Ash	CP	EE	CF
Experimental diets:									
Control Starter	8.84	5.74	23.7	3.35	3.59	6.29	26.0	3.67	3.94
Control Grower	8.91	4.99	20.7	3.38	5.83	5.48	22.7	3.71	6.40
Control Finisher	9.30	4.43	18.8	2.88	6.64	4.89	20.7	3.18	7.33
SWM Starter	8.52	5.61	23.2	6.14	6.07	6.13	25.3	6.71	6.64
SWM Grower	9.04	5.31	21.8	4.11	7.62	5.83	24.0	4.52	8.37
SWM Finisher	9.88	4.37	19.4	4.03	4.06	4.85	21.6	4.48	4.50
Feedstuff:									
SWM Pure	6.03	5.61	63.6	21.7	3.75	5.97	67.7	23.1	3.99

SWM: silkworm meal; CP: crude protein; EE: ether extract; CF: crude fiber.

**Table 3:** The in vitro digestibility of DM (DMd), CP (CPd), and OM for the samples from experiment 1—broiler chicken diets containing silkworm meal (SWM), using crude enzyme extract from broiler chickens.

	In Vitro Digestibility, %		
	DMd	CPd	OMd
Experimental diets:			
Control Starter	28.6 ^ab^	40.9 ^bcd^	26.0 ^ab^
Control Grower	27.2 ^ab^	35.5 ^ab^	24.5 ^ab^
Control Finisher	24.4 ^a^	33.7 ^a^	20.9 ^a^
SWM Starter	29.8 ^b^	45.9 ^c^	26.6 ^b^
SWM Grower	29.1 ^ab^	37.4 ^abc^	25.4 ^ab^
SWM Finisher	26.0 ^ab^	43.4 ^cd^	22.4 ^ab^
Feedstuff:			
SWM Pure	48.9 ^c^	47.7 ^d^	46.5 ^c^
SEM	1.43	1.13	1.49
*p*-value	<0.001	<0.001	<0.001

SEM: standard error of mean; DMd: dry matter digestibility; CPd: crude protein digestibility; OMd: organic matter digestibility; ^a–d^ Means in the same column with different superscript letters differ for *p* < 0.05.

**Table 4:** Correlation coefficients between the proximate composition of the experimental diets containing silkworm meal (SWM) fed to broiler chickens (samples from experiment 1) and the in vitro digestibility of DMd, OMd, and CPd.

	OMd	CPd	DM Diet	Ash Diet	CP Diet	EE Diet	CF Diet
DMd	0.999 **	0.609 **	0.894 **	0.411 *	0.914 **	0.909 **	−0.416 *
OMd		0.608 **	0.895 **	0.424 *	0.910 **	0.902 **	−0.435 **
CPd			0.433 **	0.338 *	0.468 **	0.507 **	−0.462 **
DM diet				0.521 **	0.961 **	0.949 **	−0.373 *
Ash diet					0.362 *	0.319	−0.185
CP diet						0.989 **	−0.494 **
EE diet							−0.453 **

OMd: organic matter digestibility; CPd: crude protein digestibility; DM: dry matter; CP: crude protein; EE: ether extract; CF: crude fiber; **: *p* < 0.01; *: *p* < 0.05.

**Table 5:** Proximate composition (%) of samples from experiment 2—broiler quail diets containing camelina (*Camelina sativa*) cake.

	As Is					Dry Matter			
	Moisture	Ash	CP	EE	CF	Ash	CP	EE	CF
Experimental diets:									
Control	11.3	6.48	19.7	2.02	5.50	7.30	22.1	2.28	6.20
Alan Autumn 5	11.1	6.45	22.2	2.17	6.34	7.25	24.9	2.45	7.13
Alan Autumn 10	11.7	6.27	22.1	2.89	4.49	7.09	25.0	3.27	5.08
Alan Spring 5	11.5	6.32	21.0	1.95	4.58	7.14	23.7	2.20	5.17
Alan Spring 10	11.3	5.89	20.1	2.92	4.18	6.64	22.6	3.30	4.71
Pearl Autumn 5	11.5	5.74	20.1	1.22	4.47	6.48	22.7	1.37	5.05
Pearl Autumn 10	11.4	5.99	20.5	1.69	4.72	6.75	23.1	1.91	5.33
Pearl Spring 5	11.5	5.95	20.4	1.75	4.16	6.73	23.1	1.98	4.71
Pearl Spring 10	11.1	5.87	21.2	2.30	4.27	6.61	23.9	2.58	4.80

Alan Autumn 5: diet containing 5% Alan line sowed in autumn; Alan Autumn 10: diet containing 10% Alan line sowed in autumn; Alan Spring 5: diet containing 5% Alan line sowed in spring; Alan Spring 10: diet containing 10% Alan line sowed in spring; Pearl Autumn 5: diet containing 5% Pearl line sowed in autumn; Pearl Autumn 10: diet containing 10% Pearl line sowed in autumn; Pearl Spring 5: diet containing 5% Pearl line sowed in spring; Pearl Spring 10: diet containing 10% Pearl line sowed in spring; CP: crude protein; EE: ether extract; CF: crude fiber.

**Table 6:** The in vitro digestibility of DM (DMd), CP (CPd), and OM for the samples from experiment 2—broiler quail diets containing camelina (*Camelina sativa*) cake (Alan or Pearl) for broiler quails using crude enzyme extract from 35-day-old quails.

	In Vitro Digestibility, %		
	DMd	CPd	OMd
Control	24.3	19.6	21.2
Alan Autumn 5	24.8	26.3	21.2
Alan Autumn 10	20.9	24.1	17.1
Alan Spring 5	23.1	20.9	19.2
Alan Spring 10	24.0	18.7	20.7
Pearl Autumn 5	26.2	26.7	22.9
Pearl Autumn 10	23.0	25.1	19.4
Pearl Spring 5	23.2	20.9	20.1
Pearl Spring 10	20.3	16.6	16.9
SEM	0.48	1.01	0.52
*p*-value	0.112	0.163	0.101

SEM: standard error of mean; Alan Autumn 5: diet containing 5% Alan line sowed in autumn; Alan Autumn 10: diet containing 10% Alan line sowed in autumn; Alan Spring 5: diet containing 5% Alan line sowed in spring; Alan Spring 10: diet containing 10% Alan line sowed in spring; Pearl Autumn 5: diet containing 5% Pearl line sowed in autumn; Pearl Autumn 10: diet containing 10% Pearl line sowed in autumn; Pearl Spring 5: diet containing 5% Pearl line sowed in spring; Pearl Spring 10: diet containing 10% Pearl line sowed in spring; DMd: dry matter digestibility; CPd: crude protein digestibility; OMd: organic matter digestibility.

**Table 7:** Correlation coefficients between the proximate composition (the experimental diets for broiler quails—samples from experiment 2) and the in vitro digestibility of DMd, OMd, and CPd.

	OMd	CPd	DM Diet	Ash Diet	CP Diet	EE Diet	CF Diet
DMd	0.995 **	0.551 **	0.192	−0.05	−0.15	−0.146	0.132
OMd		0.540 **	0.198	−0.08	−0.193	−0.155	0.116
CPd			0.003	0.001	0.212	−0.083	0.136
DM diet				0.271 *	−0.106	−0.055	0.774 **
Ash diet					0.449 **	0.335 *	0.688 **
CP diet						0.401 **	0.334 *
EE diet							−0.036

OMd: organic matter digestibility; CPd: crude protein digestibility; DM: dry matter; CP: crude protein; EE: ether extract; CF: crude fiber; **: *p* < 0.01; *: *p* < 0.05.

**Table 8:** Proximate composition (%) of samples from experiment 3—layer quail diets containing camelina (*Camelina sativa*) cake or camelina oil.

	As Is					Dry Matter			
	Moisture	Ash	CP	EE	CF	Ash	CP	EE	CF
Experimental diets:									
Control	10.9	10.1	19.1	0.67	14.5	11.3	21.4	0.76	16.3
Alan Autumn 15	10.8	10.4	20.3	1.54	18.1	11.7	22.8	1.72	20.3
Alan Spring 15	10.6	9.80	19.6	1.47	16.7	11.0	21.9	1.64	18.7
Pearl Autumn 15	10.2	9.97	19.8	1.98	14.8	11.1	22.1	2.21	16.5
Pearl Spring 15	10.8	9.92	20.2	2.17	14.2	11.1	22.6	2.43	16.0
Pearl Spring oil	10.9	10.4	19.3	0.22	14.0	11.7	21.7	0.24	15.7
Pearl Autumn oil	9.71	10.7	19.7	0.42	13.6	11.9	21.8	0.47	15.1

Alan Autumn 15: diet containing 15% Alan line sowed in autumn; Alan Spring 15: diet containing 15% Alan line sowed in spring; Pearl Autumn 15: diet containing 15% Pearl line sowed in autumn; Pearl Spring 15: diet containing 15% Pearl line sowed in spring; Pearl Spring oil: diet containing 0.39% Pearl line sowed in spring; Pearl Autumn oil: diet containing 0.39% Pearl line sowed in autumn; CP: crude protein; EE: ether extract; CF: crude fiber.

**Table 9:** The in vitro digestibility of DM (DMd), CP (CPd), and OM for the samples from experiment 3—layer quail diets containing camelina (*Camelina sativa*) cake (Alan or Pearl) or camelina oil (Pearl) using crude enzyme extract from 35-day-old quails.

	In Vitro Digestibility, %		
	DMd	CPd	OMd
Control	25.9 ^ab^	18.4 ^bc^	18.9 ^ab^
Alan Autumn 15	29.2 ^bc^	13.9 ^ab^	22.8 ^bc^
Alan Spring 15	29.8 ^bc^	17.8 ^bc^	23.9 ^c^
Pearl Autumn 15	27.4 ^abc^	16.8 ^abc^	21.1 ^abc^
Pearl Spring 15	24.3 ^a^	10.3 ^a^	17.7 ^a^
Pearl Spring oil	27.5 ^abc^	21.3 ^bc^	21.4 ^abc^
Pearl Autumn oil	30.6 ^c^	23.9 ^c^	24.4 ^c^
SEM	0.54	1.06	0.59
*p*-value	0.011	0.007	0.010

SEM: standard error of mean; Alan Autumn 15: diet containing 15% Alan line sowed in autumn; Alan Spring 15: diet containing 15% Alan line sowed in spring; Pearl Autumn 15: diet containing 15% Pearl line sowed in autumn; Pearl Spring 15: diet containing 15% Pearl line sowed in spring; Pearl Spring oil: diet containing 0.39% Pearl line sowed in spring; Pearl Autumn oil: diet containing 0.39% Pearl line sowed in autumn; DMd: dry matter digestibility; CPd: crude protein digestibility; OMd: organic matter digestibility; ^a–c^ Means in the same column with different superscript letters differ for *p* < 0.05.

**Table 10:** Correlation coefficients between the proximate composition (the experimental diets for laying quails—samples from experiment 3) and in vitro digestibility of DMd, OMd, and CPd.

	OMd	CPd	DM Diet	Ash Diet	CP Diet	EE Diet	CF Diet
DMd	0.993 **	0.703 **	0.364 *	0.271	−0.229	−0.355 *	0.272
OMd		0.695 **	0.376 *	0.310 *	−0.249	−0.367 *	0.310*
CPd			0.250	0.026	−0.539 **	−0.486 **	−0.154
DM diet				0.599 **	-0.305 *	−0.354 *	0.393 *
Ash diet					−0.046	−0.508 **	0.871 **
CP diet						0.674 **	0.214
EE diet							−0.184

OMd: organic matter digestibility; CPd: crude protein digestibility; CP: crude protein; EE: ether extract; CF: crude fiber; **: *p* < 0.01; *: *p* < 0.05.

**Table 11:** Characteristics of crude enzyme extract (CEX) from the proventriculus, pancreas, and duodenum of 42-day-old broiler chickens and 35-day-old quails.

	pH *	Temperature *, °C	Wavelength, λ	Enzyme Activity, U/Min/mL
Broiler chicken:				
Pepsin	3	40	660 nm	2.13
Amylase	7	37	540 nm	11.8
Cellulase	7	37	540 nm	15.3
Quail:				
Pepsin	3	40	660 nm	3.46
Amylase	7	37	540 nm	1.01
Cellulase	7	37	540 nm	10.1

* Ideal conditions for enzyme activity for a specific enzyme.

## Data Availability

The raw data supporting the conclusions of this article will be made available by the authors on request.
